# Silent Trace Eliminates Differential Eyeblink Learning in Abstinent Alcoholics

**DOI:** 10.3390/ijerph6072007

**Published:** 2009-07-20

**Authors:** Catherine Brawn Fortier, Arkadiy L. Maksimovskiy, Jonathan R. Venne, Ginette LaFleche, Regina E. McGlinchey

**Affiliations:** 1 Geriatric Research Education and Clinical Center (GRECC), VA Boston Healthcare System; Boston, MA, USA; E-Mails: amaksim@heartbrain.com (A.L.M.); jvenne@heartbrain.com (J.R.V.); 2 Department of Psychiatry, Harvard Medical School; Boston, MA, USA; E-Mail: Regina_McGlinchey@hms.harvard.edu; 3 Memory Disorders Research Center (MDRC), Boston University School of Medicine & VA Boston Healthcare System; Boston, MA, USA; E-Mail: glafleche@bu.edu

**Keywords:** alcohol, eyeblink classical conditioning, learning, discrimination, reversal

## Abstract

Chronic alcoholism has profound effects on the brain, including volume reductions in regions critical for eyeblink classical conditioning (EBCC). The current study challenged abstinent alcoholics using delay (n = 20) and trace (n = 17) discrimination/reversal EBCC. Comparisons revealed a significant difference between delay and trace conditioning performance during reversal (t (35) = 2.08, p < 0.05). The difference between the two tasks for discrimination was not significant (p = 0.44). These data support the notion that alcoholics are increasingly impaired in the complex task of reversing a previously learned discrimination when a silent trace interval is introduced. Alcoholics’ impairment in flexibly altering learned associations may be central to their continued addiction.

## 1. Introduction

Chronic misuse of alcohol leads to volume reductions in brain regions critical for associative learning using the eyeblink classical conditioning (EBCC) paradigm. First, alcohol is known to cause structural alterations in the cerebellum, a structure that is both necessary and sufficient for all forms of EBCC [1]. Such alterations have been documented by traditional post-mortem inspection [2] and more recently by *in vivo* neuroimaging studies confirming significant volume shrinkage in the cerebellar hemispheres [3].

Second, in addition to alcohol-related neuropathological changes in the cerebellum, abundant evidence from different methodologies indicates that the structural alterations due to alcohol extend into the prefrontal cortex and frontal circuitry. These are areas of the brain known to be essential for more complex or nonoptimal forms of EBCC. For example, using structural magnetic resonance imaging (MRI) Sullivan and her colleagues [4,5] have reported that each major node of the frontocerebellar circuit show volume reductions and each can be independently affected. MRI studies have also revealed greater volume losses in the frontal lobes compared to other structures [6,7]. White matter changes in alcoholics have been documented using Diffusion Tensor Imaging (DTI) [8,9]. *Post mortem* evidence from Harper [10] shows a 22% reduction in the number of neurons in the superior frontal cortex of alcoholics.

Research has demonstrated that the cerebellum is essential for all forms of EBCC (e.g., [1]). This fact, in conjunction with the known neuropathological changes to this region of the brain as the result of chronic alcohol use, lead to the prediction that abstinent alcoholics would show deficits in classical associative learning. Several studies have now demonstrated deficits in classical associative learning in abstinent alcoholics [11–14].

Cerebellar structures are critical but only part of a more extensive neural network that is involved in EBCC. Specifically, the hippocampal system and fronto-cerebellar systems are involved in more complex forms of associative learning. Importantly, whether or not the forebrain structures are essential for learning depends on the associative demands of the conditioning paradigm. Thus, while cerebellar shrinkage is the likely cause of impairment in simple forms of EBCC (i.e., single cue delay), it is unclear whether alcohol related neuropathological changes to forebrain regions, such as the hippocampal formation, frontal cortex and underlying white matter, may be responsible for the observed impairment in more complex EBCC tasks, such as trace conditioning [12] and discrimination reversal learning [11,15–18].

In the present study we examined the performance of abstinent alcoholics in EBCC tasks that require an essential contribution from forebrain structures [11,15,17,18]: delay and trace discrimination and discrimination reversal. Discrimination conditioning involves the presentation of two conditioned stimuli, one of which (CS+) is paired with an airpuff US, while the other (CS−) is presented alone (i.e., with no consequence). During the initial phase of learning, individuals do not discriminate between the CS+ and the CS− and produce CRs to both trial types. However, over additional trials, CRs to the CS− drop off and are produced, for the most part, only during the CS+ trials. Once acquisition of the discrimination occurs, the contingencies of the two CSs can be reversed. During this more complex reversal conditioning task, the significance of the two CSs is switched by making the previously paired CS+ the CS−, and the previous CS− the CS+. Importantly, this reversal occurs unbeknownst to the participants, seamlessly, and without warning. In delay conditioning the CS and the US overlap in time and terminate simultaneously. In trace conditioning, there is a silent trace period of no stimulation between the CS and the US.

Given the neuropathological evidence of cerebellar and frontal system deficits associated with alcoholism, we predicted that alcoholics’ impairments on these learning tasks would increase systematically as task difficulty increased. Alcoholics would be more impaired in reversal learning (both delay and trace) than in simple discrimination learning. Furthermore, introduction of a silent trace interval was expected to further reduce alcoholics’ ability to acquire a simple discrimination as well as reverse that discrimination as compared to delay conditioning.

## 2. Experimental Section

### 2.1. Participants

A total of 37 currently abstinent alcoholic’s (ALC) were recruited to participate in this study. All were naïve to the eyeblink classical conditioning procedures, meaning they had no prior training in eyeblink conditioning. The participants in this study were recruited from the Geriatric Research, Education, and Clinical Center (GRECC) at the Veterans Affairs Boston Healthcare System, Boston, MA, by way of distribution of flyers at local institutions, advertisements in local newspapers, and by referral from area hospitals. Abstinent alcoholic participants were screened to be free of any neurological disease or illness. Participants were also excluded for any CNS drugs, major head injury, hospitalization in a psychiatric facility > one week, or any medications for/history of severe psychiatric disorders (e.g., schizophrenia, chronic intractable obsessive compulsive disorder, agoraphobia, current major depression). History of substance abuse/dependence other than alcohol, except nicotine (current or lifetime) and cannabis (lifetime), was cause for exclusion. Cannabis use in the year prior to testing was cause for exclusion.

To meet criteria for inclusion, the abstinent alcoholic participants met one or more of the three criteria delineated below: (1) positive SMAST, (2) positive DIS-IV for DSM-IV diagnostic criteria, or (3) reported a history of ≥ 21 drinks per week for five years or longer (Oscar-Berman, personal communication). All participants were self-described alcoholics. Participants were required to have abstained from drinking for at least one month prior to participating in the study. Drinking characteristics of the sample are provided in detail below and in Table 5.

*Delay Conditioning ALCs.* Twenty abstinent alcoholic individuals were included in the delay discrimination reversal task (8 men, 12 women). The mean age of the delay ALC group was 49 years (standard deviation, SD = 8.4), the mean education in years was 13 (SD = 3.4), and the mean verbal intelligence as measured by the Wechsler Adult Intelligence Scale, Third Edition (WAIS-III) [19] was 100 (SD = 20.8).

The mean duration of abstinence prior to testing was 4.2 years (SD = 5.3), but ranged from 1 month to 19 years (see Table 1). On the Lifetime Drinking History (LDH) [20], abstinent alcoholics reported a significant history of alcohol abuse that ranged in duration from 4 years to 46 years. The mean length of abuse was 24 years (SD = 11.0). This measure yields an estimate of total lifetime exposure to alcohol using standard drink conversions (grams absolute alcohol) via two methods: (1) total lifetime drinks and (2) and total lifetime drinks controlling for weight (body weight in kg). Delay ALCs reported an average lifetime total volume of alcohol exposure of 87312 drinks or 17004 g/kg when corrected for body weight. During all drinking phases, ALCs reported a mean of 11 (SD = 7.8) standard drinks per drinking day and a mean maximum of 14 (SD = 8.7) standard drinks per drinking day. To assist in clarifying the severity of drinking across time, we also derived the average number of drinks per day consumed during reported heaviest consecutive 3-year period of drinking. The mean, for this measure, was 13 (SD = 9.2). For a profile of each alcoholic participant’s drinking history, see Table 1.

On the Self-Administered Short Michigan Alcoholism Screening Test (SMAST) [21], a self-reported measure of alcoholic behavior, ALCs reported scores ranging from 3 to 13 with a mean score of 8 (SD = 3.2). Selzer and colleagues [21] suggest that a score of 0–1 on the SMAST represents a nonalcoholic profile, a score of 2 indicates a possible alcoholic profile, and a score of 3 or higher represents an alcoholic profile.

Twelve delay ALCs met DSM-IV diagnostic criteria for Alcohol Dependence on the Diagnostic Interview Schedule for DSM-IV (DIS-IV) [22], and four met criteria for alcohol abuse. The entire 90–120 minute DIS computerized instrument was administered to participants. There were some discrepancies between the computerized measure of alcoholic behavior and participants’ self-reported and questionnaire-based history (see Table 1). It is possible that some participants had difficulty attending to the entire DIS-IV computer interview and answered unreliably during the substance abuse module, which came during the latter part of the interview. Individuals that demonstrated inconsistency between computerized DIS-IV interview and self-reported history of drinking behavior were asked to return to the laboratory for follow up DIS-IV Substance Abuse Module administration in which they answered only the 28 substance abuse related items. Four individuals were lost to follow up and the Substance Abuse Module could not be re-administered. For these participants available SMAST, LDH, and questionnaire data was used to confirm alcohol history. These four individuals reported a history consistent with alcohol abuse as defined by ≥ 21 drinks/week for a minimum of five years and self-identified as alcoholics. Two participants who did not meet criteria for alcohol abuse/dependence on the DIS-IV were classified as alcoholics on the SMAST (ALC011, ALC019; see Table 5). One of the remaining two participants with a negative diagnosis based on the DIS-IV (ALC015) reported alcohol consumption of > 20 drinks per day for over a twenty-five year period. The final participant who was lost to follow up with a negative diagnosis on the DIS-IV reported a less severe drinking history, but met criteria of ≥ 21 drinks/week for a minimum of five years and self-identified as an alcoholic (ALC004) (see Table 1).

*Trace Conditioning ALCs.* Seventeen abstinent alcoholic individuals were included in the trace discrimination reversal task (6 men, 11 women). The mean age of the trace ALC group was 51 years (standard deviation, SD = 6.6), the mean education in years was 14 (SD = 2.0), the mean verbal intelligence as measured by the Wechsler Adult Intelligence Scale, Third Edition (WAIS-III) was 104 (SD = 19.3).

The mean duration of abstinence prior to testing was 7.1 years (SD = 9.6) but ranged from 1 month to 26 years. On the Lifetime Drinking History (LDH) [20], abstinent alcoholics reported a significant history of alcohol abuse that ranged in duration from 12 years to 41 years. The mean length of abuse was 27 years (SD = 7.0). Trace ALCs reported an average lifetime total volume of alcohol exposure of 50316 drinks or 8640 g/kg when corrected for body weight. During all drinking phases, trace ALCs reported a mean of 9 (SD = 3.4) standard drinks per drinking day and a mean maximum of 15 (SD = 6.9) standard drinks per drinking day. The mean average number of drinks per day consumed during reported heaviest consecutive 3-year period of drinking was 12 (SD = 7.2). For a profile of each alcoholic participant’s drinking history, see Table 1.

Eleven trace ALCs met DSM-IV diagnostic criteria for Alcohol Dependence on the Diagnostic Interview Schedule for DSM-IV (DIS-IV) [22], and four met criteria for Alcohol Abuse. The two participants who did not meet criteria for alcohol abuse/dependence on the DIS were classified as alcoholics on the SMAST (see Table 1). As a group, trace ALCs reported scores ranging from 3 to 13 on the SMAST with a mean score of 10 (SD = 3.1) (see Table 1).

### 2.2. Procedure

Participants were brought into the laboratory individually where the examiner reviewed the informed consent form with them. Consent procedures were witnessed by an individual who was not involved with the research. All participants underwent three types of assessment: (1) Eyeblink Classical Conditioning (EBCC), (2) Assessment of Drinking, and (3) Neuropsychological Assessment. The assessments were completed in two to three testing sessions. The testing sessions were generally completed within one month for each participant. The longest interval between first and last sessions was two months. Some participants were contacted after study completion to provide additional information regarding their drinking history (see above).

*Apparatus.* The apparatus used was a modified version of that used for eyeblink conditioning in the rabbit [23,24], and one that we have used in previous eyeblink conditioning studies with humans [11,12,25,26]. Eyeblink responses were measured via surface electromyography (EMG) electrodes (Nicolet, NY) placed over the orbicularis oculi muscle of the right eye. An adjustable headband was worn to support the airpuff delivery nozzle, which delivered an airpuff to the right eye.

Data were acquired by a custom data acquisition system developed using National Instruments LabVIEW (National Instruments, Austin, TX). EMG data were acquired at 5 kHz and filtered at 2 kHz using a low pass Bessel filter. Stimulus presentation and data acquisition were controlled by custom software written in LabVIEW. The digitized EMG signal was rectified (absolute value of the amplitude) and integrated using a decay time constant of 10 ms. The integrated-rectified signal is well correlated with the eyelid closure measured using reflectance eyelid detectors [27].

**Figure 1.**
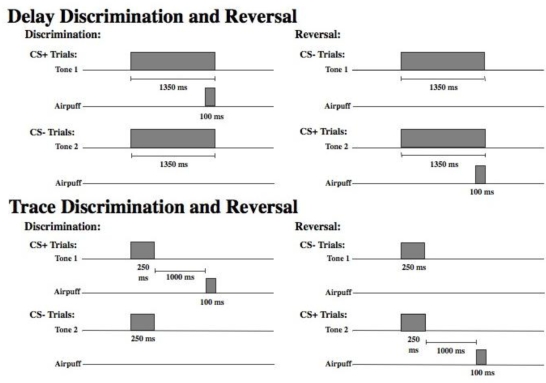
Delay and trace discrimination and reversal learning.

*Stimuli.* Figure 1 displays a schematic of the time course of each trial type in the delay and trace paradigms. As shown, there were two different tones (high and low) to signal the onset of a reinforced (CS+) or nonreinforced (CS−) trial. Assignment of the tone to these two conditions was counterbalanced across subjects. For half of the participants, discrimination learning consisted of a 1,000 Hz tone CS+ and an 85 dB, 5,000 Hz tone CS− that were delivered binaurally over headphones. The significance of the tones was reversed for the remaining participants (5,000 Hz CS+ and 1,000 Hz CS−). All other parameters remained constant. The US was presented only on CS+ trials and consisted of a 100 ms corneal airpuff that coterminated with the CS+. The magnitude of the airpuff was 3 psi for all participants. Participants were presented with 30 of each trial type randomly intermixed. Presentation of trial type was determined by computer-generated pseudo-randomized series such that no more than three reinforced or nonreinforced trials could occur in succession. During reversal learning, the CS− became the CS+, and the CS+ became the CS−. The transition from discrimination training to reversal training was seamless and uninterrupted. Participants were again presented with 30 trials of each type randomly intermixed. A total of 120 EBCC learning trials were presented including 60 discrimination trials and 60 reversal trials, half reinforced (CS+) and half nonreinforced (CS−). In delay conditioning the CS was 1350 ms in duration and the CS and the US overlapped in time and terminated simultaneously. In trace conditioning, the CS was 250 ms in duration and there was a silent trace period of 1000 ms between the CS and the US (see Figure 1).

*Neuropsychological Assessment.* All study participants received a neuropsychological test battery that targeted cognitive domains affected by alcoholism (tasks sensitive to frontal and cerebellar dysfunction) and those thought to underlie the learning and expression of classical conditioned responses in associative learning tasks including executive function, motor function, and memory. A test of general verbal intelligence was also administered. Verbal abilities were assessed with Wechsler Adult Intelligence Scale, Third Edition (WAIS-III) [19]. Memory/medial temporal function was assessed with the Wechsler Memory Scale, Third Edition (WMS-III) [19] and the Warrington Word and Facial Recognition test [28]. Executive/frontal system function was assessed with the Trailmaking test [29], Controlled Oral Word Association test (COWAT) [30], Wisconsin Card Sorting test (WCST) [31], Stroop Color-Word test [32], and Ruff Figural Fluency test [33]. Motor/cerebellar function was assessed with the Grooved Pegboard [34], Finger Tapping test [35,36], and an Ataxia Battery [37].

*EBCC Procedure.* Each participant underwent an audiology screening using a model 119 Beltone portable audiometer. The criteria of Solomon [38] was employed and participants whose threshold in either ear was greater than 15 dB above normal (40 dB) were excluded. However, all participants’ thresholds fell within the normal range and thus none of the participants recruited for this study were excluded based on results of the audiology screening. Participants were seated in an upright chair and the examiner fitted them with the eyeblink apparatus. Throughout the session, the experimenter sat in the same room, out of the direct view of the participant and answered questions as they arose. Each conditioning session consisted of a total of 120 conditioning trials. Prior to the onset of each trial, there was a 750 ms baseline recording period. The inter-trial interval averaged 10 seconds, but varied randomly from 8 to 12 seconds.

*Definitions.* An eyeblink was only scored as a CR if its amplitude was at least four standard deviations greater than the mean baseline response amplitude. Eyeblinks with latencies less than 100 ms following CS onset were recorded as alpha responses and not considered CRs [39].

## 3. Results and Discussion

There were no significant differences between the alcoholic groups run in the delay and trace paradigms in regard to age, education, or VIQ (p’s > 0.45). Furthermore, the groups were matched for drinking history. There were no significant differences between groups on any of the drinking measures including DSM-IV diagnosis, SMAST, or each LDH quantification of lifelong drinking behavior measure (see Table 1, p’s > 0.15).

The primary measures of interest were the percentage of conditioned responses acquired during CS+ and CS− trials. Other dependent variables examined included characteristics of both the conditioned and unconditioned responses: CR onset latency, CR peak latency, CR amplitude, and UR amplitude. CR onset latency refers to the time at which the CR amplitude first reached four standard divisions above baseline. Alternatively, CR peak latency represents the time at which the given CR reached its highest amplitude. CR peak latency likely captures the level of adaptiveness of a CR (optimally, a CR will peak just before the onset of the airpuff). The CR amplitude is measured as peak amplitude and refers to the amount of EMG muscle activity during a CR. UR amplitude is measured as peak amplitude and refers to the amount of EMG muscle activity during the UR period, and reflects the unconditioned reflex in response to the airpuff.

### 3.1. Discrimination Learning

#### Conditioned Response Acquisition

**Figure 2.**
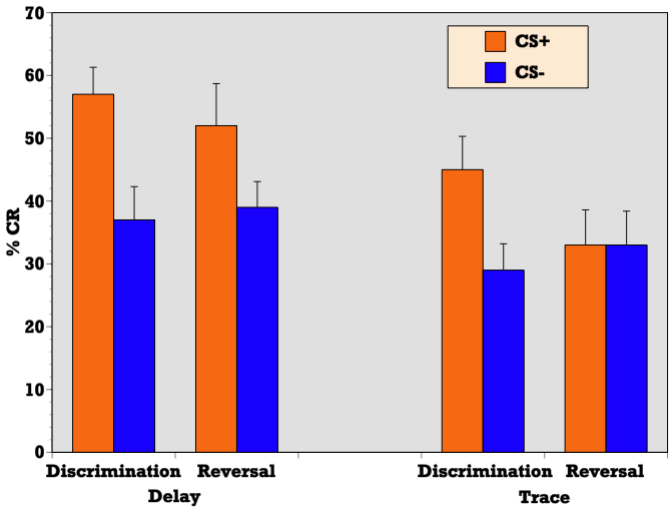
CR Acquisition. Mean percentage conditioned responses (CRs) for reinforced (CS+) and nonreinforced (CS−) trials during delay and trace conditioning.

Independent samples T-test confirmed a significantly greater percentage of CRs during reinforced trials as compared to nonreinforced trials during delay discrimination learning (t = 4.32, p = 0.001) indicating that abstinent alcoholics (ALC) were able to respond differentially on CS+ versus CS− trials and acquire the initial discrimination in a delay paradigm (see Figure 2). ALCs produced a CR on 58 (SE = 4.3) percent of CS+ trials and 37 (SE = 5.1) percent of CS− trials during delay conditioning. Similarly, a t-test confirmed a significantly greater percentage of CRs during reinforced trials as compared to nonreinforced trials during trace discrimination learning (t = 3.32, p = 0.004) indicating that abstinent alcoholics (ALC) were also able to respond differentially on CS+ versus CS− trials and acquire the initial discrimination in the context of a trace paradigm (see Figure 2). ALCs produced a CR on 45 (SE = 5.3) percent of CS+ trials and 29 (SE = 4.2) percent of CS− trials during trace conditioning.

T-test demonstrated a significant difference between the delay and trace paradigms in the mean percentage of CRs acquired on reinforced trials during discrimination learning (t = 2.04, p = 0.05) indicating that although alcoholics were able to acquire the initial discrimination during both delay and trace paradigms to some degree, participants produced more CRs on reinforced trials during delay discrimination learning than trace discrimination learning (see Figure 2).

#### Difference Scores

Difference scores were calculated by subtracting the mean percentage of CRs during nonreinforced trials from the mean during reinforced trials (Difference Score = %CRs on CS+ trials minus %CRs on CS− trials). Alcoholics’ difference score during delay discrimination learning was 21 (SE = 4.9). Alcoholics’ difference score during the trace discrimination learning was 16 (4.8). T-test on the mean differential learning scores revealed there was no significant difference between delay and trace differential CRs during discrimination learning (t = 0.786, p = 0.437).

#### Learning Curves

**Figure 3.**
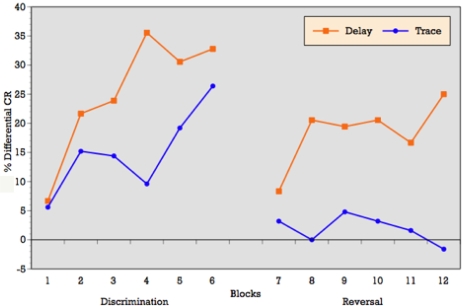
Learning Curves: Conditioning trials were collapsed into six blocks of five trials each. Difference Scores were calculated for each block by subtracting the mean percentage of CRs during nonreinforced trials from the mean during reinforced trials (Difference Score = %CRs on CS+ trials − %CRs on CS− trials).

As can be seen in Figure 3, when conditioning trials were collapsed into six blocks of five trials each, the ALC participants demonstrated an overall increase in the percentage of CRs across the six discrimination learning blocks of reinforced trials during delay conditioning, peaking at block 4 and remaining moderately steady across blocks 5 and 6. T-tests on mean percentage of CRs acquired block by block confirmed significant differences between blocks 1 and 3 (p = 0.01), blocks 1 and 4 (p = 0.001), blocks 1 and 6 (p = 0.002), and marginal significance between blocks 1 and 5 (p = 0.06) during delay conditioning. The learning curve for trace conditioning was similar, although acquisition was not as strong as in trace conditioning. T-tests confirmed significant differences between blocks 1 and 2 (p = 0.01), blocks 1 and 3 (p = 0.007), blocks 1 and 4 (p = 0.005), blocks 1 and 6 (p = 0.005), and marginal significance between blocks 1 and 5 (p = 0.06).

Block by block comparisons between paradigms revealed a significant difference in CR production during reinforced trials at block four (t = 2.49; p = 0.02). Percentage of CRs on nonreinforced trials remained stable across learning blocks in both paradigms. Block by block comparisons showed no difference in CR production during nonreinforced trials between paradigms. Overall, Figure 3 reveals that alcoholics attained some level of differential learning during both delay and trace discrimination, although acquisition was greater during delay conditioning.

#### Response Latency & Amplitude

Independent samples T-tests of CR response latency during reinforced trials revealed that none of the measures differed significantly between paradigms (p’s > 0.07) during discrimination learning (see Table 2). Similarly, there were no differences between paradigms for CR or UR amplitude during discrimination learning (p’s > 0.50) (see Table 2).

### 3.2. Reversal Learning

#### Conditioned Response Acquisition

During reversal learning, the previously reinforced CS+ became the CS−, and the previously nonreinforced CS− became the CS+ requiring participants to flexibly alter their previously learned stimulus contingencies, decreasing their CR production to the old CS+ and increasing CR production to the new CS+.

T-test confirmed a significantly greater percentage of CRs during reinforced trials as compared to nonreinforced trials during delay reversal learning (t = 3.01, p = 0.007) indicating that abstinent alcoholics (ALC) were able to respond differentially on CS+ versus CS− trials and acquire the reversal of stimulus contingencies in the context of a delay paradigm (see Figure 2). ALCs produced a CR on 53 (SE = 6.3) percent of CS+ trials and 39 (SE = 3.9) percent of CS− trials during delay conditioning. However, a t-test revealed that during trace conditioning, the percentage of CRs during reinforced trials as compared to nonreinforced trials did not significantly differ (t = 0.139, p = 0.891) indicating that abstinent alcoholics (ALC) were unable to reverse the previously learned discrimination in the context of a trace paradigm (see Figure 2). ALCs produced a CR on 33 (SE = 5.6) percent of CS+ trials and 33 (SE = 5.4) percent of CS− trials during trace conditioning.

T-test demonstrated a significant difference between the delay and trace paradigms in the mean percentage of CRs acquired on reinforced trials during reversal learning (t = 2.27, p = 0.03) indicating that participants produced more CRs on reinforced trials during delay reversal learning than trace reversal learning (see Figure 2).

#### Difference Scores

Difference scores were calculated by subtracting the mean percentage of CRs during nonreinforced trials from the mean during reinforced trials (Difference Score = %CRs on CS+ trials − %CRs on CS− trials). Alcoholics’ difference score during delay reversal learning was 16 (SE = 4.8). Alcoholics’ difference score during the trace was 0.6 (SE = 4.2), indicating that alcoholics were unable to achieve differential learning during the trace reversal task. This was confirmed by a t-test on the mean differential learning scores, in which there was a significant difference between delay and trace differential CRs during reversal (t = 2.08, p = 0.045).

#### Learning Curves

As can be seen in Figure 3, when conditioning trials were collapsed into six blocks of five trials each, the ALC participants demonstrated relatively flat production in the percentage of CRs across the six reversal learning blocks of reinforced trials during delay conditioning, peaking at blocks 3, 4 and 6. T-tests on mean percentage of CRs acquired block by block showed no significant differences across learning blocks during delay reversal learning. Despite the flat curve, there is evidence of some increased acquisition over trials with rapid learning in the first block. The learning curve for trace conditioning was less consistent and showed no evidence of acquisition across blocks and no evidence of rapid acquisition in block 1 as seen in the delay paradigm. T-tests on mean percentage of CRs acquired block by block confirmed no significant differences across learning blocks during trace reversal learning.

Block by block comparisons between paradigms revealed a significant difference in CR production during reinforced trials at block 2 (t = 2.24; p = 0.03), block 4 (t = 2.20; p = 0.04), block 5 (t = 2.16; p = 0.04), and marginal significance at block 1 (t = 1.95; p = 0.06). Percentage of CRs on nonreinforced trials remained stable across learning blocks in both paradigms. Block by block comparisons showed no difference in CR production during nonreinforced trials between paradigms. Overall, Figure 3 reveals that alcoholics attained some level of differential learning during delay reversal learning, but no acquisition of differential CRs during trace reversal learning.

#### Response Latency & Amplitude

Independent samples t-tests of CR response latency during reinforced trials revealed that none of the measures differed significantly between paradigms (p’s > 0.12) during reversal learning (see Table 2). Similarly, there were no differences between paradigms for CR amplitude during reversal learning (p’s > 0.10) (see Table 2). There was, however, a significant difference in UR amplitude between delay and trace conditioning on reinforced trials during reversal learning (t = 3.31; p = 0.002) (see Table 3). Consequently, to ensure that the differences observed in acquisition were not confounded by a difference in unconditioned reflex to the airpuff, UR amplitude was entered as a covariate in an analysis of covariance (ANCOVA) of the mean percentage of CRs acquired on reinforced trials (paradigm as the between subjects variable). This analysis indicated that reversal UR amplitude was not a significant covariate (F = 0.772; p = 0.386).

#### Alpha Responses

The number of short latency alpha responses did not differ between the groups (p’s > 0.4). The mean number of alpha responses across all trial types for the ALCs was 17 (SE = 4.95) and 19 (SE = 4.54) for the control participants.

### 2.3. Drinking Severity, EBCC Learning, and Neuropsychological Function

Examination of measures of drinking severity and their relation to associative learning and cognitive function was performed. Post-hoc analyses of drinking severity as assessed by the DIS revealed a difference on the mean CR acquisition during reversal learning (DIS 1 vs 2: t = 2.12; p = 0.043), indicating that individuals meeting criteria for alcohol dependence performed worse than those meeting criteria for abuse. Specifically, individuals meeting criteria for alcohol dependence were unable to acquire CRs differentially during the more complex reversal learning phase (Mean Reversal Difference Score = 4.20, SD = 3.71), whereas individuals meeting criteria for alcohol abuse were able to acquire CRs differentially during reversal (Mean Reversal Score = 21.67, SD = 9.19). There were no significant differences in learning performance among those that did not meet criteria for abuse or dependence on the DIS (DIS = 0) and those that did meet criteria for abuse or dependence.

Correlational analyses of neuropsychological tests and measures of drinking severity are reported in Table 4. Several tests of memory function were found to significantly correlate with measures of drinking severity (see Table 4). These included subtests of the Wechsler Memory Scale, Third Edition assessing verbal and visual immediate and delayed memory as well as Warrington recognition memory for faces. Several tests of executive function were also found to significantly correlate with measures of drinking severity including total perseverations during the verbal fluency task and performance on the Stroop task. Eta-squared is also provided in Table 4. The total lifetime volume of alcohol consumed, as measured by the Lifetime Drinking Questionnaire, explains approximately 35 percent of the variance in performance on the Stroop Interference Trial, a task of inhibition.

Correlational analysis of neuropsychological tests and measures of EBCC learning performance are reported in Table 5. One test of memory function was found to significantly correlate with EBCC performance: visual reproduction immediate recall raw and scaled scores. This measure was significantly correlated with discrimination learning (p’s = 0.01, see Table 5). One motor measure was found to significantly correlate with EBCC: composite score for walk-on-line from the ataxia battery (p = 0.01). Eta-squared is also provided in Table 5. The composite ataxia measure explains approximately 30 percent of the variance in production of CRs on reinforced trials during discrimination learning.

## 4. Conclusions

The primary finding from this study is that alcoholics are unable to acquire differential learning as task difficulty increases from the delay to trace paradigm. A limitation of the study is that a normal control group of nonalcoholic individuals was not included. However, we feel the study is still important to our understanding of the cognitive deficits related to chronic alcoholism because it demonstrates a relative decline in alcoholics’ performance as task demands become more complex. Notably, we have already shown that abstinent alcoholics are impaired in the simpler delay discrimination reversal task when compared to a normal group [11]. In this earlier study, alcoholics did acquire some level of differential responding during both discrimination and reversal learning, but it was impaired compared to normal controls. Thus, the evidence of some differential learning in a delay paradigm when compared to a control group, coupled with the current results showing no differential learning in a trace paradigm, clearly demonstrates that as task difficulty increases and learning demands are more complex (e.g., a silent trace interval is introduced), abstinent alcoholics’ ability to reverse a previously learned discrimination is eliminated.

The ability to reverse a learned discrimination has been linked by both animal and human studies to forebrain structures, in particular the hippocampal system [15,17] and prefrontal cortex [18]. The reversal impairment following prefrontal lesions and thalamic mediodorsal nucleus lesions in non-human animals involved slowed acquisition of reversal contingencies. Rabbits were able to acquire the reversed discrimination but more slowly than normal animals [16,40]. The selective impairment in reversal learning in abstinent alcoholics is therefore in line with the animal literature [18,41]. Given alcohol’s documented neurotoxic predisposition for cerebellar and frontal brain regions, the current study also lends support for the notion that a cerebellar-thalamic-prefrontal cortex module controls eyeblink associative learning during nonoptimal conditions or more complex tasks such as reversal learning [42,43]. However, it is possible that alcohol may exert a neurotoxic effect on the hippocampal system as well, which could explain these findings at least in part.

Interestingly, the nature of the reversal learning impairment observed in abstinent alcoholics appears to be different than that associated with hippocampal system damage. The abstinent alcoholics’ reversal deficit appeared to be due to an inability to produce a normal percentage of conditioned responses during CS+ trials rather than a deficit in inhibiting responses to new CS− (i.e., extinguishing the old CS+) as seen with hippocampal damage. Alcoholics appear to have a selective impairment in the ability to flexibly manipulate previously learned associations. In particular, they are impaired in producing a positive response to a previously acquired neutral or inhibited response. We therefore conclude, similarly to a recent study in our laboratory [11], that frontal system damage, as seen in alcoholics, disrupts the ability to differentially respond at a high rate to a stimulus previously responded to at a low rate.

The frontal system dysfunction related to chronic alcoholism may have behavioral consequences related to alcoholic relapse. Frontal system dysfunction may further perpetuate alcoholics’ inability to maintain abstinence because the previously learned behavioral patterns, such as drinking triggers and maladaptive coping mechanisms, are so pervasive that they interfere with the individuals’ ability to flexibly learn new patterns of behavior. This idea is similar to Hyman’s hypothesis [44] that addiction represents “a pathological usurpation of neural processes that normally serve reward-related learning.” (p. 565) and may help provide a framework of understanding what happens in alcoholics’ resistant to treatment.

Correlations and *post-hoc* analyses revealed significant differences in some measures of basic learning acquisition between individuals grouped according to severity of alcohol dependence. Individuals meeting criteria for alcohol dependence on the DIS performed worse than those meeting criteria for abuse. Specifically, individuals meeting criteria for alcohol dependence were unable to acquire differential responding during the more complex reversal-learning phase, whereas individuals meeting criteria for alcohol abuse were able to acquire CRs differentially during reversal. This indicates that more severe levels of alcoholism can lead to impairments in acquisition, particularly during more complex, demanding learning tasks.

In selecting the neuropsychological test battery for this study, the domains of memory, executive function, and motor function were chosen based on empirically-driven hypotheses regarding neurologic sequela of alcoholism. The relation between neuropsychological test performance and drinking severity was supported by correlational analyses (see Table 4). Executive function (perseverative behavior on the verbal fluency task and speed and inhibition on the Stroop task) appeared to be particularly sensitive to measures of drinking severity. Memory function including verbal and visual immediate and delayed memory as well as facial recognition memory were related to multiple measures of drinking severity. Correlations between motor measures and drinking measures were expected given the documented effect of alcohol on the cerebellum, but were not observed in this small group of alcoholics. These findings vary somewhat from others in the literature. Sullivan [45,46] reported deficits in the domains of executive function (male alcoholics only), verbal and nonverbal working memory (female only), visuospatial function (male and female), and motor function (male and female) in abstinent alcoholics in her examination of neuropsychological function in a large group of male and female alcoholics. Sullivan and colleagues also document preserved declarative memory function [45,46] as well as recovery of short-term memory function with maintained sobriety [47] in alcoholics. However, findings of impaired memory performance in abstinent alcoholics are common (e.g., [48]. It is also important to note that only one measure of verbal memory was related to drinking severity in this sample. The preponderance of relationships between memory performance and severity of drinking were in the visual domain. Therefore these results may reflect relationships between drinking severity and visuospatial processing more than visual memory *per se*. This warrants further scrutiny in subsequent investigations.

It is important to note that our abstinent alcoholic group may include individuals with less significant drinking histories than often studied (e.g., alcoholics with recent hospitalized detoxification). Our primary objective was to include a wide range of alcoholic profiles representative of alcoholism in a community setting. We attempted to avoid over-sampling a more severe subgroup of alcoholics such as those alcoholic individuals in clinical treatment settings [49]. Significant differences in EBCC learning were observed in this group, demonstrating that community-dwelling abstinent alcoholics have deficits in complex, nonoptimal classical conditioning learning paradigms.

The relationship between neuropsychological test performance and measures of EBCC learning (see Table 5) supported the hypothesized neural circuit underlying the formation of new memory traces in classical conditioning of associative relationships. Specifically, we predicted that the cerebellar-thalamic-prefrontal cortex module, as defined by Weiss and Disterhoft [42], supported eyeblink associative learning in the discrimination reversal tasks. Neuropsychological test performance in the domain of motor function was most strongly related to EBCC learning performance. Given the known cerebellar contribution to EBCC, it was not surprising that a measure of motor function was correlated with discrimination learning. As anticipated, memory function as assessed by neuropsychological test performance was also correlated with EBCC measures. We also expected tasks of executive function to be related to EBCC learning, and particularly to the more complex task of reversal learning as observed in our previous investigation [11]. This was not the case in this small sample. Although largely exploratory given the small sample size, these findings indicate that cerebellar and medial temporal function as assessed by neuropsychological tests are related to discrimination reversal learning, particularly in a trace paradigm.

In conclusion, the current study examined simple discrimination and reversal learning in the context of both delay and trace learning paradigms. As task difficulty increased from the delay to trace paradigm and a silent trace interval was introduced, abstinent alcoholics were unable demonstrate any differential learning. These findings indicate that alcoholics’ ability to learn and acquire new associations becomes more pronounced when there is a temporal gap between relevant information/stimuli. Alcoholic addiction may result from the over-learning of pathological, persistent associative memories or associative learned responses that interfere with the ability to learn new, more adaptive associations. This interference in new learning is more pronounced when there are gaps in time between the presentation of new information and previously learned information. As a result, alcoholic individuals are prone to relapse based on their patterns of learning. Gaps of time between new, adaptive learning and old, pathological learning likely exacerbate relapse to previous behavioral patterns.

## Figures and Tables

**Table 1.** Drinking characteristics of the abstinent alcoholics. ALC001 – ALC020 were run in the delay paradigm. ALC021 – ALC037 were run in the trace paradigm. Diagnostic Interview Schedule for DSM-IV (DIS-IV) [22]: Alcohol Dependence = 2, Abuse = 1, No Diagnosis = 0. Self-Administered Short Michigan Alcoholism Screening Test (SMAST) [21]: 0–1 = Nonalcoholic Profile, 2 = Possible Alcoholic Profile, and ≥3 = Alcoholic Profile. Lifetime Drinking History (LDH) [20] drinking descriptors are presented for all drinking phases. The LDH is designed to aggregate all drinking phases across the lifespan. Therefore this instrument assesses all time periods (not just phases of heavy drinking) in which a participant reported using alcohol regardless of quantity of use. Note three ALCs were not administered SMAST (lost to follow up). Means and standard deviations (SD) are provided.

	**Years of Abuse**	**Months sober**	**DIS**	**SMAST**	Total Lifetime Drinks (g/kg)*	**Average Drinks per day**	**Maximum Drinks per day**	**Total Lifetime Drinks**	**3-Year Heaviest Drinking: Average Drinks per day**

ALC001	12	12	1	6	1536	10.00	20.00	7680	10
ALC002	21	24	2	10	6828	7.14	11.93	39108	8
ALC003	32	12	2	4	4020	6.00	6.00	23928	6
ALC004	35	1	0	.	4752	4.50	7.00	28800	6
ALC005	33	36	2	13	9192	8.63	10.88	62640	8
ALC006	27	6	2	11	32952	17.33	18.17	170700	20
ALC007	25	3	2	12	33264	21.00	25.00	189000	21
ALC008	40	24	1	4	5316	2.75	4.75	29976	5
ALC009	28	84	2	5	9444	11.48	13.68	66492	22
ALC010	12	36	1	9	3972	10.00	10.00	17340	8
ALC011	15	180	0	5	1656	5.00	7.00	7200	5
ALC012	18	228	2	3	3888	7.00	12.00	24984	8
ALC013	31	108	1	5	3924	3.00	3.25	13068	5
ALC014	4	84	2	10	1080	6.00	10.00	8640	6
ALC015	30	3	0	.	50436	21.00	22.00	227556	21
ALC016	5	6	2	9	2508	21.00	21.00	10080	21
ALC017	46	48	2	12	139404	32.50	40.00	651600	40
ALC018	22	5	2	9	12192	9.20	15.60	67440	12
ALC019	25	6	0	8	11880	15.00	17.60	90432	22
ALC020	22	108	2	.	1776	4.75	7.25	9552	8


**Mean**	**24.15**	**50.70**	**1.40**	**7.94**	**17004**	**11.16**	**14.16**	**87312**	**13.00**
**SD**	**11.00**	**63.62**	**0.82**	**3.21**	**31644**	**7.81**	**8.70**	**147816**	**9.21**


ALC021	18	264	2	12	26628	17.25	30.25	164616	33
ALC022	26	3	1	11	14160	8.00	19.25	72576	10
ALC023	21	18	2	12	12936	9.60	14.40	58440	10
ALC024	12	312	2	9	6084	10.00	17.50	27600	10
ALC025	26	7	2	10	4440	4.50	6.25	22608	6
ALC026	24	192	2	11	3888	7.60	14.00	24048	10
ALC027	29	48	1	11	7092	15.00	23.00	55056	24
ALC028	31	144	2	12	9960	10.00	19.29	72840	21
ALC029	24	4	0	9	8064	6.80	9.80	37524	8
ALC030	36	300	2	13	17796	10.00	19.00	92160	10
ALC031	41	1	2	3	9036	7.33	10.83	50736	10
ALC032	23	3	1	3	3468	8.50	25.83	20256	10
ALC033	28	134	0	7	3156	4.00	9.50	15216	4
ALC034	33	6	2	9	6396	8.60	11.60	53424	10
ALC035	26	6	2	6	2628	7.00	8.33	23808	15
ALC036	23	3	1	12	5172	5.43	7.86	31536	12
ALC037	35	4	2	12	5976	6.28	9.57	32952	9


**Mean**	**26.82**	**85.24**	**1.53**	**9.53**	**8640**	**8.58**	**15.07**	**50316**	**12.00**
**SD**	**7.04**	**115.08**	**0.72**	**3.10**	**6252**	**3.40**	**6.90**	**36696**	**7.19**

* adjusted for weight

**Table 2.** Conditioned Response Measure Means (standard deviation) and Unconditioned Response Measure Means (standard deviation).

	**CR Onset Latency**		**CR Peak Latency**		**CR Amplitude**		**UR Amplitude**	
**Discrimination**	CS+	CS−	CS+	CS−	CS+	CS−	CS+	CS−
**Delay** **Trace**	880 ms (89) 851 ms (231)	802 ms (283) 936 ms (96)	1,006 ms (61) 916 ms (249)	894 ms (313) 981 ms (94)	19 mV (15) 17 mV (14)	16 mV (17) 16 mV (10)	45 mV (16) 44 mV (16)	12 mV (12) 10 mV (9)
**Reversal**	CS+	CS−	CS+	CS−	CS+	CS−	CS+	CS−
**Delay** **Trace**	900 ms (106) 865 ms (247)	903 ms (91) 790 ms (305)	1,015 ms (75) 933 ms (268)	990 ms (63) 860 ms (333)	14 mV (8) 16 mV (12)	13 mV (5) 19 mV (18)	56 mV (20) 37 mV (15)	10 mV (5) 10 mV (9)

**Table 3.** Unconditioned Response Measure Means (standard deviation).

	**UR Amplitude**	

Discrimination	CS+	CS−
Delay	45 mV (16)	12 mV (12)
Trace	44 mV (16)	10 mV (9)
Reversal	*CS+*	*CS−*
Delay	56 mV (20)	10 mV (5)
Trace	37 mV (15)	10 mV (9)

**Table 4.** Correlational analyses revealed significant (p ≤ 0.01) correlations between neuropsychological tests and alcohol consumption.

**Neuropsychological Test**	**Drinking Measure**	**Pearson Correlation**	**R^2^**	**Significance**
**Memory / Medial Temporal**				
WMS-III				
Verbal Paired Associate II Raw Score	LDH Total Volume Average Drinks/Day	−0.455 −0.441	0.207 0.195	0.009 0.011
Visual Reproduction I Raw Score	LDH Total Weight Corrected LDH Total Volume Average Drinks/Day	−0.473 −0.475 −0.493	0.224 0.226 0.243	0.008 0.008 0.006
Visual Reproduction II Raw Score	LDH Total Weight Corrected LDH Total Volume Average Drinks/Day Maximum Drinks/Day	−0.486 −0.485 −0.584 −0.467	0.236 0.235 0.341 0.218	0.007 0.007 0.001 0.009
Visual Reproduction II Scaled Score	Average Drinks/Day	−0.494	0.244	0.006
Warrington Facial Recognition	LDH Total Weight Corrected LDH Total Volume Average Drinks/Day Maximum Drinks/Day	−0.462 −0.482 −0.543 −0.484	0.214 0.232 0.295 0.234	0.010 0.007 0.002 0.007
**Executive / Frontal**				
Verbal Fluency Total Perseverations	Length of Abuse (years) LDH Total Weight Corrected LDH Total Volume	0.547 0.589 0.573	0.299 0.347 0.329	0.002 0.001 0.001
Stroop Color-Word T-score	LDH Total Weight Corrected LDH Total Volume	−0.466 −0.465	0.217 0.216	0.009 0.010
Stroop Interference T-score	LDH Total Weight Corrected LDH Total Volume Average Drinks/Day	−0.581 −0.590 −0.524	0.338 0.348 0.275	0.001 0.001 0.003

**Table 5.** Correlational analyses revealed significant (p ≤ 0.01) correlations between neuropsychological tests and measures of EBCC learning.

**Neuropsychological Test**	**EBCC Learning Measure**	**Pearson Correlation**	**R^2^**	**Significance**
**Memory / Medial Temporal**				
WMS-III Visual Reproduction I Raw Score Visual Reproduction I Scaled Score	Discrimination Score Discrimination Score	0.445 0.453	0.198 0.205	0.014 0.012
**Motor / Cerebellar**				
Composite Ataxia Measure Walk on line, walk heel-to-toe arms folded across chest eyes open and closed	Discrimination % CR+	−0.524	0.294	0.011
